# The Role of Natural Killer (NK) Cells in Dengue Virus Infection: A Narrative Review

**DOI:** 10.1002/hsr2.72783

**Published:** 2026-07-08

**Authors:** Farjana Islam

**Affiliations:** ^1^ Department of Biochemistry and Molecular Biology Shahjalal University of Science and Technology Sylhet Bangladesh

**Keywords:** ADCC, cytokine production, DENV, inhibitory receptors and activating receptors, natural killer cells

## Abstract

**Background and Aims:**

In the innate immune response, natural Killer (NK) cells have a significant role in protection against viral infections, including the Dengue virus (DENV). NK cells identify and eliminate virus‐infected cells through diverse mechanisms, such as cytokine production, cytolytic activity, and antibody‐dependent cellular cytotoxicity (ADCC), thereby contributing to reducing viremia and regulating adaptive immunity in the context of DENV. This review summarises the biological mechanisms of NK‐cell‐mediated immune response against DENV, describes the viral evasion of NK immune protection and explores the insights for the immunotherapeutic approaches and drug development.

**Methods:**

To conduct this narrative review, we searched the literature on NK cell activation, receptor expression, cytokine responses, virus evasion strategies, and experimental or emerging therapeutic approaches in DENV.

**Results:**

NK cells recognise DENV through the interaction of inhibitory receptors along with the human leukocyte antigen (HLA) class I molecules, which in turn strengthens the ADCC response. DENV can apply virus evasion strategies for modulating NK cell‐mediated immunity and reducing the antiviral effect. In addition, impaired and exhausted NK cell phenotypes are associated with the progression and severity of dengue syndromes.

**Conclusion:**

NK cells play a double‐edged sword in protective response and immune function during DENV. A deeper understanding of their NK cell mechanism, subset heterogeneity, functional response, and evasion strategies governs key insights for vaccines and therapeutic strategies that prompt antiviral immune response.

## Summary

- NK cells have a significant role in the front line of the protective immune response through cytokine production, cytolytic activity and antibody‐dependent cellular cytotoxicity.
- NK cells recognise dengue virus through the interaction of inhibitory receptors with HLA.
- Understanding the NK cells' immune response to DENV will give insights into therapeutic strategies and drug development.

## 1 Introduction

The transmission of Dengue virus (DENV) is considered a global health issue around the globe, especially in tropical and subtropical regions. It has been estimated that approximately 400 million people have been infected with DENV each year [1]. The dengue virus consists of four distinct subtypes DENV‐1 to DENV‐4, which are transmitted to humans via bites of infected mosquitoes [2, 3]. DENV belongs to Flavivirus genus in the Flaviviridae family and also encompasses several other pathogens, such as the yellow fever virus, West Nile virus (WNV), Japanese encephalitis virus (JEV), and Zika virus (ZIKV) [4]. Infection with the same serotype can exhibit lifelong immunity in response to the same serotype. Secondary infection with a different serotype can cause severe sickness due to antibody‐dependent enhancement (ADE) [5]. DENV lead to a broad range of clinical manifestations, from asymptomatic issues and mild sickness with fever to severe dengue hemorrhagic fever (DHF) and dengue shock syndrome (DSS). DHF and DSS patients may experience a high mortality rate of up to 20% [6].

While adaptive immunity plays a significant role in defence, the innate immune response elicits a frontline protection against DENV. Viral RNA in infected cells are detected via the action of pattern recognition receptors (PRRs) such as Toll‐like receptors, including TLR3, TLR7, TLR8, RIG‐I‐like receptor (RLR), and Melanoma Differentiation‐Associated gene 5 (MDA5) that induce signalling pathways such as NF‐κB and IRF3/7, consequently interferon production [7, 8, 9]. Infected cells induce an antiviral state in neighboring cells, followed by the upregulation of interferon‐stimulated genes (ISGs). The binding of Type I interferons (IFNs) to IFN receptors triggers the activation of natural killer (NK) cells, which activates the JAK‐STAT signalling pathway, which may impact the pathogenesis of severe dengue infection [10].

As part of the innate immune system, NK cells mediate the role of pathogenesis against DENV through the production of cytotoxic proteins, such as perforin and granzymes, to mediate the apoptosis of the infected cells [11]. NK cells also secrete cytokines like IFN‐γ and tumour necrosis factor‐alpha (TNF‐α) to elicit antiviral effects during the initial phase of infection [12, 13]. Although the immune protective role of NK cells is complex, it involves both detrimental and beneficial impacts. The initial activation of NK causes viral clearance and protection. Uncontrolled or malfunctioning NK cell activation can disproportionate disease severity by triggering endothelial damage, inflammation and extreme vascular permeability, which are key points of severe illness [14]. Therefore, NK cells play a double‐edged sword in DENV infection, where their antiviral effect must be counterbalanced against their significant role in mediating immunopathogenesis. However, the pinpoint role of NK cells during DENV infection remains controversial, as it has been reported to elicit both protective antiviral effects and contribute to immunopathogenesis depending on the disease severity. This culminates in the need for a more comprehensive understanding of NK cell function in different clinical contexts. This review aims to give an understanding of the earlier activation of NK cells upon DENV infection. It also emphasizes their protective role against DENV infection and explores the possibility of acting as therapeutic targets.

## 2 Methods

This narrative review is conducted on a broader and structured data search by using electronic database systems such as PubMed, Scopus, Embase, Web of Science and Google Scholar. This database search was carried out by using keywords such as “natural killer cells”, “dengue virus”, “NK cell activation”, “NK receptor interaction”, “cytokine responses”, “ADCC and ADE”. It mainly focuses on peer‐reviewed articles published as original research articles and clinical investigations. Studies mainly emphasize NK cell biology, front‐line immune response to DENV, NK cell activation and induced cytokine response upon DENV infection, DENV‐mediated immune evasion strategies and host‐pathogen association were included. Publications that are not directly related to NK cell biology in DENV were excluded. Although this study does not maintain systematic review arrangements, efforts were made to make a more balanced and broader representation of the recent research.

## 3 Functional Heterogeneity of NK Cell Subsets

NK cells consist of diverse populations with distinct functional subsets, not homogeneous subsets. NK cell subsets, such as CD56^bright and CD56^dim, play distinct roles among circulating NK cells. CD56^bright NK cells have a slight cytotoxic activity, but are significant in cytokine production, immune surveillance, and immune interaction with other lymphocytes [15]. CD56^dim subsets elicit stronger cytotoxic activity and are the potential mediators of killing DENV‐infected cells directly. Upon DENV infection, their activation, expansion and functional activities might be different depending on the serotypes, stages and severity of disease [16, 17].

However, findings regarding the prevalence of NK cell subsets during DENV infection, which remain controversial, suggest that it may vary depending on the subset‐specific responses and be influenced by viral replication and host immune status. However, the circulating compartment represents one subset of the heterogeneous NK cell population. Apart from that, tissue‐resident NK cells are resident in the liver, lung and uterus. Tissue‐resident NK cells are distinguished by distinct phenotypic marker expression, transcriptional gene and functional activities surrounded by the local microenvironment [18]. Furthermore, peripheral circulating NK cells play a predominant role in tissue homeostasis, tissue remodelling, and local immune responses, whereas tissue‐resident NK cells do not [19]. Importantly, these subsets differ significantly in their distribution and activation stimuli, as well as in physiological and pathological conditions within the blood and tissues [20]. As a consequence, analyses that depend exclusively on peripheral blood may offer a restricted and potentially skewed perspective on NK cell biology, failing to adequately represent subset‐specific responses and neglecting significant functions carried out by tissue‐resident populations [21]. Acknowledging this diversity is crucial for the precise interpretation of NK cell responses in DENV infection. It is important to note that the most recent evidence is focused on peripheral blood samples, which do not clearly reveal the functional participation of tissue‐resident NK cells. Most current evidence is derived from peripheral blood analyses, which may not accurately reflect the functional contributions of tissue‐resident NK cells. This demonstrates a key weakness and broader active controversy in understanding NK cell biology upon DENV infection.

## 4 NK Cell Activation in the Earlier Onset of Dengue Infection

NK cells are pivotal components of the innate immune system, inducing rapid and effective defence against viral infections, including DENV [12]. NK cells act in the first line of antiviral defence to detect and kill diseased cells, to reduce viral replication and viremia, which contributes to the disease progression and severity [12]. In DENV, the immune activation of NK cells is associated with the balance between activating and inhibitory receptors. In the initial onset of dengue infection, NK cells express higher levels of activation markers (CD69, CD38, HLA‐DR) and cytotoxic factors such as perforin and granzyme B to enhance cytolytic activity [22]. The strength of NK cell activity is restricted by the expression of inhibitory receptors like KIRs and NKG2A interacting with self‐MHC class I molecules on healthy cells [12, 23]. The regulatory mechanism can be dysregulated upon DENV, influencing the downregulation of MHC Class I expression or upregulation of stress‐induced signals such as MICA/B and ULBPs. It consequently activate the NKG2D receptor [22]. NK cell immune response has been triggered by these “missing self” or “induced self” signals. In turn, it allows the expression of activating receptors like NKp30, NKp44, and NKp46, assisting in the identification and destruction of dengue‐infected cells [24]. Cytokines such as Interleukin‐12 (IL‐12), IL‐15, IL‐18, and type I IFN‐α/β are produced in the initial stage of infection, revealing a significant role in priming and activating NK cells. Clinical studies demonstrate that efficient protection against viral infections and weaker disease outcomes are associated with the strength and efficiency of NK cell activation (Table 1) [12, 22]. In contrast, dysregulation or exhaustion in NK cell phenotype is linked to the exacerbation of viremia and more severe disease outcomes [29]. Therefore, NK cells play a crucial role in the early containment of the dengue virus and in coordinating immune responses that affect disease progression and protection.

**Table 1:** Summary of clinical studies representing NK cell responses and association with dengue disease severity. Initially, balanced NK activation (cytotoxicity and IFN‐γ) is associated with protection, whereas dysregulated or exhausted NK responses are linked to severe disease.

Study/Year	Cohort/Sample	NK markers/functions assessed	Key findings	Clinical correlation
Azeredo et al. [25]	42 patients (Brazil)	Flow cytometry: CD69, perforin, granzyme B	Elevated NK activation markers during acute infection.	Stronger NK activation in mild dengue than in severe cases.
Petitdemange et al. [12]	30 acute dengue patients	NK phenotyping, cytokine assays, transcriptomics	Skin‐homing (CLA^+^) NK cells activated; upregulated IFN‐γ signaling.	Initial activation linked with milder disease and viral clearance.
Sun et al. [26]	Ex vivo ADCC/ADE functional work with patient sera	NK depletion/add‐back, ADCC assays, IFN‐γ blocking	NK cells reduce ADE in PBMC assays; IFN‐γ important mediator	Functional NK/IFN reduced ADE → protective role; NK dysfunction increases ADE
Zimmer et al. [27]	Cohort of acute dengue patients, phenotyping & trafficking	High‐dimensional flow, transcriptional profiling	NK cells activated and primed for tissue homing early after symptom onset	Reveals temporal dynamics & tissue‐trafficking program of NKs in patients.
Laoprasopwattana et al. [28]	ADCC assays using patient plasma	ADCC functional assays with purified NKs/targets	Plasma mediates ADCC; NKs are effectors in ADCC against DENV	Indicates NK‐mediated ADCC contributes to control and is measurable in patient plasma.

Abbreviations: ADCC, antibody‐dependent cellular cytotoxicity; ADE, antibody‐dependent enhancement; DENV, dengue virus; IFN‐γ, interferons‐gamma; NK; PBMC, peripheral blood mononuclear cells.

## 5 The Interplay Between Antibody‐Dependent Cellular Cytotoxicity (ADCC) and ADE

ADCC, one of the crucial immune mechanisms that bridges the gap between innate and adaptive immunity, facilitates the removal of infected or transformed cells. Although other immune cells, like macrophages, neutrophils, and eosinophils, are involved in this regulation, NK cells primarily strengthen the immune responses [30]. In ADCC, the immune response is facilitated by the interaction of effector cells, such as NK cells, and antibodies coated with target cells via Fc receptors, which in turn enhance the activation and cytotoxic response [31]. The ADCC effector mechanism is mainly initiated by IgG antibodies attached to viral antigens interacting with NK cells expressing FcγRIII (CD16), followed by the identification of the specific Fc region of these antibodies [32]. It results in the activation of NK cells, mediating the release of cytotoxic granules such as perforin and granzymes, which stimulate apoptosis of the antibody‐coated target cells [33]. During dengue viral infection, ADCC appears to have a key role in the elimination of infected cells and controlling the viral replication, although this mechanism has a dual nature [34]. An exacerbated or uncontrolled ADCC immune activity can turn into immunopathology and tissue damage, while moderate ADCC activity aids in the pathogenesis of DENV. Furthermore, in dengue infections, the presence of cross‐reactive, non‐neutralising antibodies from prior infections can promote ADE, a pivotal immunopathological action associated with disease severity. Upon secondary virus infection of heterologous serotype, the antibodies generate immune complexes with the virus without virus neutralisation, which promotes the virus load into immune cells, such as monocytes, macrophages, and dendritic cells that possess Fc receptors [5]. This process, in turn, results in the amplification of infected cells and virus replication. This increased uptake of the infected cells through the Fcγ receptors would lead to more infected cells, hence increased levels of viremia [16]. ADE is categorized into two groups: extrinsic ADE promotes the virus particles' entry into the immune cells, and intrinsic ADE induces the management of a virus‐friendly microenvironment for the enhancement of viral replication and suppression of protective response [5]. Furthermore, ADE is associated with exacerbated immune activation, such as excessive cytokine production and extreme vascular leakage, creating severe disease outcomes such as DSF and DSS. This hyperactivation may also include the activation of the complement system and the inflammatory cascades, which would lead to endothelial dysfunction [35]. Consequently, the severity is influenced by the association between protective ADCC and detrimental ADE [36]. Although ADCC helps in the clearance of the virus through Fc‐mediated mechanisms, such as cytotoxicity and phagocytosis, this may not be the case if the antibodies are of poor quality and instead induce ADE [37]. It is noteworthy that the levels of antibodies, their affinities, and their specificities are critical in determining the type of antibody response, which could be either protective or enhancing, with the risk of ADE being the highest at sub‐neutralising levels [5]. In general, ADCC appears to be a significant effector function of strengthening humoral immunity, neutralisation and phagocytosis and highlighting a key level of immune protection against DENV. This, therefore, highlights the dual role of antibodies in the context of dengue virus infection, where the immune response could be either protective or pathogenic. The interrelationship between ADCC and ADE is not fully clear, and it is still unclear whether the ADCC immune response can counteract ADE in vivo. This relationship appears to be more complicated due to the variation in antibody quality, concentration and Fc receptor interaction.

## 6 Cytokine‐Mediated Protective Immune Response

Cytokines serve a significant role in controlling the immune system's response to DENV, mediating both protective functions and enhancing pathogenesis. Innate immune cells, such as dendritic cells, macrophages, monocytes, and NK cells, tend to release cytokines for inducing an antiviral response in the initial stage of DENV. Pattern recognition receptors (PRRs), including TLRs and RLRs, enhance the secretion of type I interferons (IFN‐α and IFN‐β), followed by the identification of viral RNA [38, 39]. This consequently activates the JAK‐STAT signalling pathway, resulting in the upregulation of ISGs, which limit viral replication [40]. Cytokines also mediate the bridge between innate and adaptive immunity. Proinflammatory cytokines, such as TNF‐α, IL‐12, and IL‐18, influence NK cell cytotoxicity and IFN‐γ production, in turn enhancing the management of viral replication [41]. Together, these cytokines induce immune responses, assist in restricting viremia and enhance adaptive immune response. However, imbalanced or excessive cytokine production increases the chance of dengue severity, while similar cytokines elicit protective and disease severity depending on their distribution and prevalence.

Increased serum levels of TNF‐α, IL‐6, IL‐8, IFN‐γ, and IL‐10 are closely correlated with DHF and DSS [42, 43]. TNF‐α and IL‐6 tend to increase vascular permeability and endothelial activation, influencing plasma leakage and shock syndrome, while IL‐8 exacerbates inflammatory conditions.

In terms of anti‐inflammatory properties, excessive IL‐10 production can reduce antiviral immune response and extend the duration of viral clearance [44]. In addition, non‐structural proteins, such as NS2A, NS4B, and NS5, encoded by DENV, deteriorate interferon signalling, limiting the upregulation of antiviral genome and assisting in immune evasion [45]. Therefore, while cytokines mediate significant antiviral defence, their dysregulation underlies the key point of immunopathology related to severe disease outcomes. Understanding these immune responses is pivotal for the development of drug and therapeutic approaches that induce protective cytokine effects while controlling inflammatory conditions.

## 7 Viral Evasion of NK Responses

During viral infections, NK cells facilitate earlier actions through the cytolytic activity and cytokine release to restrict the replication of the viral gene. DENV has constructed diverse mechanisms to escape or evade immune response mediated by NK cells. The suppression in viral activation is achieved through the upregulation of HLA (MHC class I) molecules on virus‐infected cells, which consequently interact with inhibitory receptors on NK cells. Laboratory studies of cells harboring dengue virus non‐structural replicons reveal heightened surface expression of HLA class I, raised transcription of components involved in antigen processing (such as TAP and LMP), and decreased vulnerability to NK cell‐mediated lysis due to enhanced binding to inhibitory receptors (like KIRs and LIR‐1) on NK cells [22, 46]. Human studies conducted both in vivo and ex vivo indicate that during acute dengue infection, various cell types (including monocytes) exhibit increased levels of total HLA class I. Experimentally blocking HLA Class I on DENV‐infected monocytes can significantly boost NK cell degranulation. However, blocking HLA‐E (a type of nonclassical Class I) produced a lesser effect [47]. Another strategy for evasion is the disruption of type I IFN production and signalling. Non‐structural proteins of DENV, specifically NS2A and NS4B, inhibit the secretion of IFN‐β by interfering with pathways involving RIG‐I/MDA5‐MAVS/TBK1/IKKε and obstructing the phosphorylation processes of TBK1 and IRF3. The DENV NS2B/3 protease also interacts with IKKε, hindering the activation of IRF3. These actions diminish the cytokine environment necessary for the complete activation of NK cells [48, 49]. There are indications that the viral NS1 protein has dual functions. While antibodies specific to NS1 can activate NK cells through ADCC, NS1 may also inhibit interferon‐stimulated genes in certain viral variants, thereby reducing signalling that supports NK cell activity [50, 51]. In conclusion, DENV non‐structural proteins can evade immune response from NK cells by upregulating inhibitory ligands (classical HLA‐I). DENV also modifies nonclassical HLA molecules (like HLA‐E) suppressing interferon production mediated by non‐structural proteins, and impacting antibody‐mediated NK activation. Understanding these evasion strategies could aid in the development of vaccines or therapies aimed at restoring NK cell functionality.

## 8 Dengue Infection Is Influenced by Host Genetic and Immunological Factors

The clinical outcomes of DENV are influenced not only by viral factors but also by host genetic and immunological factors. Some disease outcomes appear to be severe DHF or DSS, while most infections remain asymptomatic or mild. Differences in immune regulation among individuals influence disease severity and outcomes. In addition, genetic factors reveal a significant role in the determination of virus susceptibility and disease progression. Antigen presentation and T cell responses differ through the variation in the expression of human leukocyte antigen (HLA) genes. Certain alleles, such as HLA‐A24 and HLA‐DRB10901, are linked to disease progression, while HLA‐B57 and HLA‐A33 are associated with a protective immune mechanism [52]. Additionally, polymorphisms in MICB and MICA are crucial because they impact the MICB and MICA proteins, which serve as ligands for NK cell receptor NKG2D, and can induce NK cell activation and initial antiviral defence [53]. Other important genetic loci, including those encoding DC‐SIGN (CD209), TNF‐α, and IFN‐γ, are considered pivotal because of their contribution to the enhancement of immune response, such as viral entry, inflammation, and cytokine production, and consequently influence the outcome of the disease [54]. ADE can happen, followed by the secondary infection with a different serotype. In this case, sub‐neutralising or non‐neutralising antibodies allow the entry of the virus into Fcγ receptor‐expressing cells, which can lead to the virus amplification and increase the production of inflammatory cytokines [55]. Furthermore, cross‐reactive memory T cells developed from previous infections may cause alternation in cytokine profiles. It results in excessive immune activation and vascular leakage, a phenomenon referred to as “original antigenic sin” [56]. Moreover, variations in innate immune signalling, including IFN pathways and pattern recognition receptors, can impact viral load or cytokine production. Mutations in IFNAR1 or TLR3 induce weaker antiviral responses and strengthen disease severity [57]. In summary, the pathogenesis of DENV arises from complex interactions between host genetic variations and immune responses, balancing protective immunity with immunopathology. Gaining knowledge about these mechanisms is essential for developing targeted therapeutics and vaccines against dengue. However, the relative significance of internal versus immune functions in understanding disease severity persists as difficult to distinguish because those elements act in a complicated and context‐dependent manner.

## 9 Implications for Vaccine and Therapeutic Development

NK cells are one of the essential components of the innate immune system that guide early defence against viral infections, including DENV. They succumb to infected or disease cells through cytotoxic mechanisms and produce antiviral cytokines such as IFN‐γ, which assist in the modulation of viral replication and shaping adaptive immunity. Understanding how the innate immune response of NK cells is influenced by DENV infection makes a significant contribution to both vaccine design and therapeutic development (Table 2).

i. Evidence‐based insights: Experimentally established evidence suggests that NK cells become activated and play a crucial role in viral clearance during acute DENV infection. Virus evasion strategies, including upregulation of inhibitory HLA class I molecules, downregulation of activating ligands, and inhibition of type I interferon signalling, result in reduced NK cell cytotoxicity and less cytokine production [12, 47]. In addition, type I interferon and IL‐15 signalling pathways appear as a pivotal driver of triggering NK cell activation and robust adaptive immunity.
ii. Emerging evidence: Emerging evidence suggests that NK cells may exhibit features of trained immunity or memory‐like responses. It, in turn, induces an immune response during secondary infection; however, this is not fully elusive upon DENV infection [59]. Similarly, live attenuated vaccines such as CYD‐TDV (Dengvaxia) and TAK‐003 have strengthened the innate immune activation, although their direct contribution to NK cell functionality is still unclear and remains inferred [60, 61].
iii. Future directions and therapeutic potential: Future therapeutic and vaccine strategies may facilitate promoting the strength of NK cells after using cytokine adjuvants (e.g., IFN‐α, IL‐15) or monoclonal antibodies engineered for advanced ADCC. These strategies have the capability for virus clearance while minimizing ADE. Although these strategy‐based applications during DENV infection remain theoretical, requiring further lab‐based validation.

**Table 2:** Summary of NK cell–based strategies in DENV infection, including their mechanisms of action, level of evidence, and relevance for forming vaccines and therapeutics. Evidence levels are grouped as experimental/clinical, emerging, or inferred (requiring further validation) in DENV.

Study/Year	Strategy	Mechanism	Evidence level	Relevance
Beltran and Lopez‐Verges [22]; Piersma and Brizic [38]	Type I interferon signalling	Mediates NK activation and protective response	Strong (experimental)	Central to innate antiviral response
Mishra et al. [58]; Piersma and Brizic [38]	IL‐15 stimulation	Promotes NK proliferation and survival	Strong (experimental)	Main cytokine for NK cell homeostasis
Petitdemange et al. [12]	NK cell activation in DENV	Cytotoxicity and IFN‐γ production	Strong (clinical/experimental)	Associated to antiviral effect
McKechnie et al. [47]; Petitdemange et al. [12].	Virus evasion of NK cells	Inhibition via HLA‐I	Strong (experimental)	Reduces NK cell strength
Neely et al. [59]	NK memory‐like responses	Promotes secondary responses	Moderate (emerging)	Limited DENV‐specific data
Lee et al. [60]; Parra‐Gonzalez et al. [61]	Dengue vaccines (Dengvaxia, TAK‐003)	Elicits innate immune response	Limited (indirect)	NK‐specific effects unclear
Chen et al. [62]; Sun et al. [26]; Thomas et al. [63]	ADCC‐enhancing antibodies	Enhances NK‐mediated cytotoxicity via Fc receptor	Moderate (other viruses)	Well studied in HIV and influenza. Limited validation in DENV
—	Cytokine adjuvants (IFN‐α, IL‐15)	Promotes NK activation	Hypothetical (DENV)	Require future experimental validation

Abbreviations: ADCC, antibody‐dependent cellular cytotoxicity; DENV, dengue virus; IFN‐γ, interferons‐gamma; IL‐15, interleukin‐15; NK, natural killer.

Thus, considering NK cell modification in the development of dengue vaccines and therapeutics demonstrates an optimistic strategy for the protection and efficient disease control without the risk of immunopathology, including ADE.

## 10 Research Gap and Future Directions

Despite significant progress in understanding NK cells' role in DENV infection, several important research gaps remain. Current studies mainly focus on peripheral blood NK cells. The dynamics and functions of tissue‐resident NK cells, especially in the liver and spleen, which are key sites for DENV replication, are poorly understood. Moreover, the exact ways DENV affects NK cell receptor expression and signalling to avoid immune detection are not fully clear. The balance between protective and harmful NK responses is also uncertain. This is particularly true regarding how NK cell‐derived cytokines contribute to vascular leakage and severe dengue symptoms.

Future research should use single‐cell transcriptomics and high‐dimensional immune profiling to detail NK cell differences during various stages of infection. Longitudinal studies that combine viral load, immune activation markers, and clinical outcomes are necessary to establish cause‐and‐effect links between NK activity and disease severity. Additionally, examining NK cell memory‐like responses in DENV infection may demonstrate advancements in long‐term immunity. Addressing these research gaps will be crucial for gaining a more advanced understanding of NK cell biology in response to DENV infection and for designing further immunological studies. Considering these issues will be pivotal to rectifying the current debates and developing the implementation research.

## 11 Conclusion

NK cells create a linkage between innate and adaptive immunity during DENV infection, performing key antiviral and immunoregulatory functions. The strength of NK cells is finely tuned by the receptor signalling and cytokines, inducing antiviral effects. However, chronic antigenic stimulation, the immune escape strategies of DENV, and altered inflammatory conditions can lead to dysfunction or exhaustion in NK cell phenotypes. In addition, NK cell‐mediated antibody‐dependent cellular cytotoxicity can aid in the clearance of DENV‐infected cells. But, in some instances, antibody‐dependent enhancement may significantly participate in disease progression. In summary, NK cells serve effector and modulator functions in protection against DENV, while also influencing inflammatory conditions when immunoregulatory systems fail. Importantly, the dual protective and pathogenic roles of NK cells persist as a debatable issue in DENV immunology that needs further validation to govern therapeutic applications. Understanding the mechanisms that govern NK cell activation and dysregulation demonstrates advanced opportunities for the formulation of therapeutic interventions and vaccines that prompt antiviral defences without weakening immune‐associated damages.

## Author Contributions

**Farjana Islam:** conceptualization, writing – original draft, review and editing. The author has fully read and approved the final version of the manuscript.

## Funding

The author have nothing to report.

## Conflicts of Interest

The author declares no conflicts of interest.

## Transparency Statement

Farjana Islam affirms that this manuscript is an honest, accurate, and transparent account of the study being reported.

## Data Availability

No data was used for the research described in the article. Data sharing not applicable to this article as no datasets were generated or analyzed during the current study.
